# Mental Health of Parents as Caregivers of Children with Disabilities: Based on Japanese Nationwide Survey

**DOI:** 10.1371/journal.pone.0145200

**Published:** 2015-12-21

**Authors:** Yui Yamaoka, Nanako Tamiya, Yoko Moriyama, Felipe Alfonso Sandoval Garrido, Ryo Sumazaki, Haruko Noguchi

**Affiliations:** 1 Department of Health Service Research, Faculty of Medicine, University of Tsukuba, Tsukuba-shi, Ibaraki, Japan; 2 Department of Health and Welfare Service, National Institute of Public Health, Wakou-shi, Saitama, Japan; 3 Faculty of Political Science and Economics, Waseda University, Shinjuku-ku, Tokyo, Japan; 4 Department of pediatrics, Faculty of Medicine, University of Tsukuba, Tsukuba-shi, Ibaraki, Japan; Sun Yat-sen University, CHINA

## Abstract

The number of children with disability is increasing gradually in Japan. Previous researches in other countries have reported that parents as caregivers (CGs) of children with disability have mental health problems, but the actual situation has not been examined nationwide in Japan so far. The aim of this study was to evaluate the association between mental health of CGs who had children with disability and characteristics of children, CGs, and household based on the nation-wide survey. This study utilized data from 2010 Comprehensive Survey of the Living Conditions, and defined children with disability aged 6 to 17. Individual data of children and CGs were linked, and 549 pairs of them were extracted. The Japanese version of Kessler 6 (K6) was used to assess mental health status of caregiver, scored 5 and over represented to general psychological distress. Logistic regression was used to evaluate the associations of interest. The almost half (44.4%) of CGs had psychological distress (k6 score; 5 +) in nationwide, and 8.9% of CGs might have serious mental illness (K6 score; 13+). After adjusting covariates of child, CG, and household factors, CG having a current symptom (OR, 95% CI: 3.26, 1.97–5.39), CG's activity restriction (OR, 95% CI: 2.95, 1.38–6.32), low social support (OR, 95%CI: 9.31, 1.85–46.8), three generation family (OR, 95% CI: 0.49, 0.26–0.92), and lower 25% tile group of monthly household expenditure (OR, 95% CI:1.92, 1.05–3.54), were significantly associated with psychological distress of CGs. This study encourages health care providers to pay more attentions toward parent's mental health, especially for in case of having low social support, and lower income family. Further research should examine the detailed information of child's disease and disability, medical service use, and quality and quantity of social support in nationwide to straighten the system for supporting services of both children with disabilities and their CGs.

## Introduction

The number of children with disabilities is gradually increasing in Japan. In 2011, there were 225,000 children with physical disability who received “Physical Disability Certificate (*Shintai Syougaisya Tecyou*)” or intellectual disabilities who received “Mental Disability Certificate (*Ryouiku Tecyou*)” living at home in Japan, accounting for 1.1% of the total population of persons under 18 years of age [1, 2]. Physical Disability Certificate includes person with visual impairment, hearing impairment, speech difficulty, physically disabled, internal organ disorders including respiratory disorder, cardiac problems, and so on. Mental Disability Certificate targets person with intellectual disorder that found less than 18 years old.

Over five years, from 2006 to 2011, the percentage of children with disabilities rose by 7%, whereas the total population of persons under 18 years of age decreased by 4% in Japan [1, 2]. Furthermore, pervasive developmental disability has gained increasing attention recently. Teachers reported that 6.5% of children in primary schools experience strong difficulties in learning and behavior (i.e., hyperactive or impulsive tendency) despite the absence of an intellectual disability [3]. The possible reason for increasing the number of children with disability varies depends on each disease or disability. For example, the mortality rate of infants who were born with extremely low birth weight (500–1,000g) has been improving year by year from 55.3% in 1980 to 15.2% in 2000 [4]. Follow-up survey for children with extremely low birth weight at age of six showed that 16.8% of them had cerebral palsy and 20.3% of them had intellectual disability [5]. Furthermore, average maternal and paternal age of having a first child have become later gradually in Japan: 30.6 years old for mothers and 32.6 years old for fathers in 2014 [6]. Previous research reported that higher age of mothers at birth was related to increase of child with intellectual disability [7], and higher age of fathers at birth was associated with an elevated risk of high-functioning autistic-spectrum disorder [8]. However, despite of types of disability, parents usually play essential roles as primary caregivers. Although becoming a caregiver of a child is an unexpected ‘career’ for parents [9], caring for a child with a disability can impose multiple strains on the parents due to health problems [10–12], financial costs [13–15], time demands, and hindrance of social participation, such as a working career [16, 17]. For example, Montes & Halterman estimated that families with a member that has Autism spectrum disorder experienced a 14% loss of annual income, or a loss of $6,200. Furthermore, family composition has gradually shifted over recent decades, and there are more nuclear, single-parent, and families with working mothers than before [18]. Nuclear family accounted for nearly 80% of all household with children, and 60% of mothers had a job in 2011 [18]. Among household with children less than six years old, paternal involvement for housekeeping or child rearing is scarce compared to mothers: only 12 minutes per week for housekeeping and 39 minutes per week for child rearing, comparing to mothers who spent 3.6 hours for housekeeping and 3.4 hours for child rearing on average [19]. In these situations, mothers sometimes undertake responsibility for caregiving to a child with disability in fewer supports from family members.

Under these difficult situations of raising a child with a disability, parents tend to experience mental health problems. For example, elevated levels of depressive symptoms were seen among mothers of children with epilepsy [20] or children with developmental disabilities [21], and higher psychological distress was reported among parents of children with pervasive developmental disorders [22].

Furthermore, mental health problems of parents may place children at risk for adverse health outcomes. Multiple previous studies have demonstrated that maternal depression was significantly associated with child behavioral and emotional problems [20, 23], or lower health-related quality of life [20]. Depressed mothers were reported to show negative parenting behavior [24], and lower quality of supervision [25]. Therefore, it is important issue to evaluate parental mental health toward better health outcome of children with disability.

Previous studies conducted in Japan have examined mental health of caregivers with General Health Questionnaire [26], caregiving burden with Japanese version of the Zarit Caregiver Burden Interview [27, 28], or psychological distress with Kessler 6 (K6) scale[22]. However, these studies targeted limited study populations, such as parents of children with disabilities from one to several medical facilities or patients’ associations. Therefore, this study aimed to examine the mental health of parents (hereafter referred to as caregivers; CGs) of children with disabilities based on nationally representative data in Japan using K6 which has been widely implemented in many countries [29] to assess psychological distress.

## Materials and Methods

### Study population

The present study utilized data from the ‘health questionnaire’ and ‘household questionnaire’ of the Comprehensive Survey of Living Conditions: CSLC (*Kokumin Seikatsu Kiso Chousa in Japanese*) in 2010 [18], which was a nationwide cross-sectional survey conducted by the Ministry of Health, Labour and Welfare in June 2010. The sampling method of CSLC was based on enumeration districts (ED) from Census. The entire land of Japan is divided into 982,000 EDs, and each ED includes approximately 50 households. CSLC in 2010 utilized EDs from Census in 2005, and randomly selected 5,510 EDs [18]. CLSC in 2010 surveyed 289,363 households, and collected questionnaires from 229,785 households, which covered 609,019 household members. The respondents were all household members, except for individuals who were hospitalized or institutionalized during the survey period. All questionnaires were conducted by self-administration, as long as the respondent as at least 6 years old. For those who were less than 6 years old, next-of-kin responded to the questionnaire. The response rate was 79.4% of all households.

**Fig 1:**
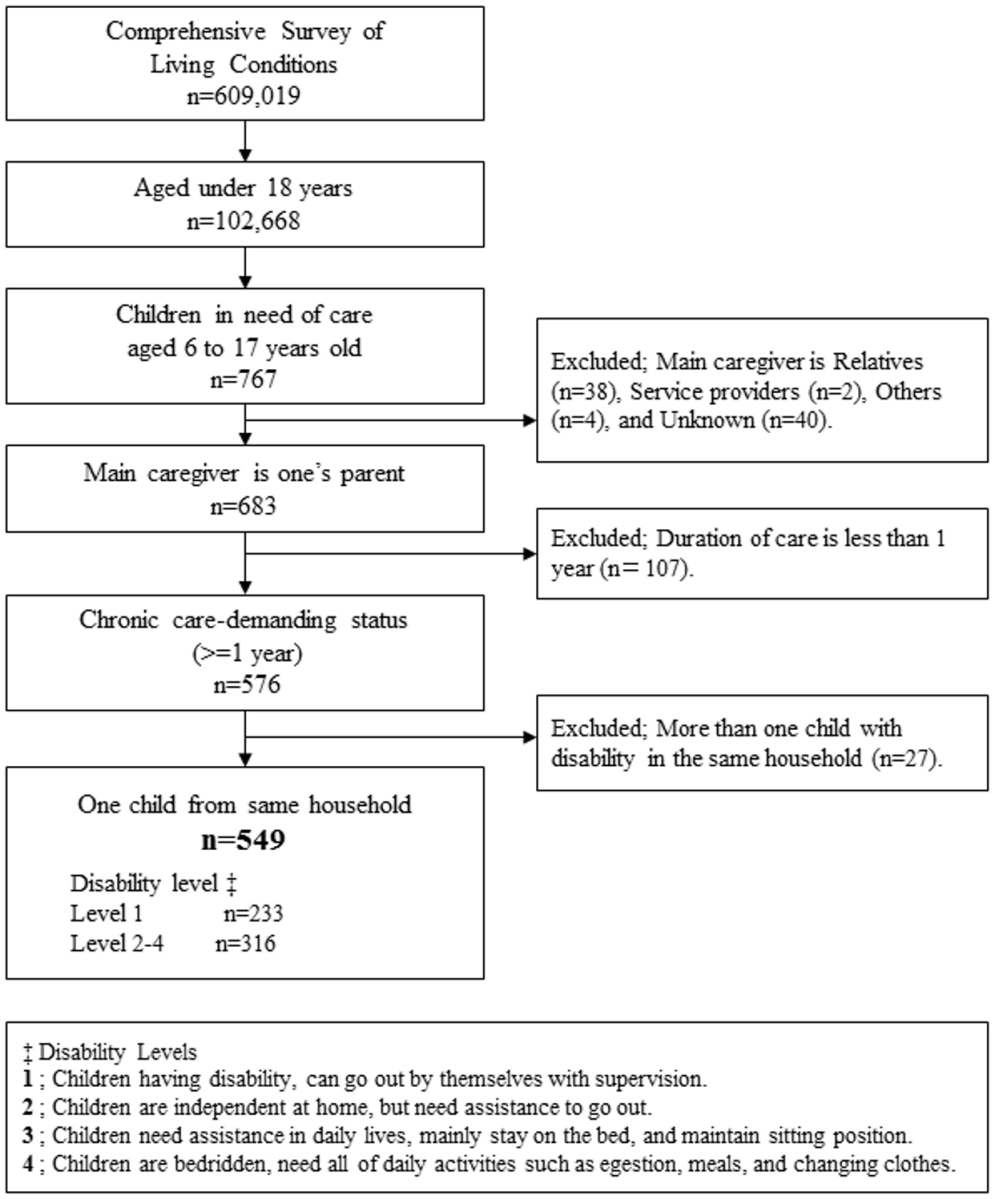
Flow chart of Study Sample.

The flow chart (Fig 1) indicates how samples were extracted for our quantitative analysis. First, we extracted ‘children’, defined as persons under 18 years of age, from the entire sample of the CSLC (n = 102,668). Second, to detect children with disabilities and their main caregivers (CGs), we examined the responses to the question, “Does he/she need assistance or supervision?” (n = 767). This question was asked for children aged 6 and over. Third, we only included children whose main CGs were their own parents (n = 683) because this study was focused on the mental health status of parents. Fourth, children who had received care for less than one year, and these children were excluded from our study sample to avoid including children who suffered from acute injury or disease (N = 576). As the severity of disability, we categorized care-demanding status into four levels. Level 1 indicates that a child has a disability but can go out by oneself, and children with level 1 status was defined as mild disability (n = 233). We included children with care levels 2 to 4 (n = 316) as moderate to severe disability, which were defined as follows: level 2, ‘the child is independent at home but needs assistance to go out (level 2)’; level 3, ‘the child needs assistance in activities of daily life and mainly stays in bed in a sitting position’; and level 4, ‘the child is bedridden and requires assistance with all activities of daily life, such as egestion, meals, and changing clothes’. Fifth, we selected one child who had more severe disability or child who was youngest when multiple children with disability lived in the same household, and excluded non-corresponding 27 children. Finally, we merged child (care-recipient)-based data with parent (main CG)-based data according to the question that identified the main CG for each child within a household. Conclusively, we included 549 dyads of children (233 children with mild disability, 316 children with moderate to severe disability) and their parents in our study population.

### Mental health measures

We used the Japanese version of the Kessler 6 (K6) scale of psychological distress as an outcome variable to assess mental health status of the CGs. Kessler and his colleagues developed 6-item short screening instruments to ask respondents how frequently they experienced symptoms of non-specific psychological distress during the past 30 days. Non-specific psychological distress is a widespread indicator of mental health and is the core dimension that people with a wide range of mental disorders have typically experienced [30]. The K6 has been widely implemented in many countries [29], and the Japanese version of the K6 has been validated [31]. All six items are examined using a 5 point scale (0–4), with the total score thus ranging from 0 to 24. Kessler et al. [30] originally developed K6 to have good precision in the 90th-99th percentile range of the population distribution of non-specific psychological distress. The K6 showed excellent performance in detecting 30-days Diagnostic and Statistical Manual of Mental Disorders 4th Edition (DSM-IV) mood and anxiety disorders among community respondents. For detecting DSM-VI mood and anxiety disorders, the Areas under Receiver operating characteristics (ROC) curves (hereafter, it called as AUC) was 0.94 with K6 in interview survey [31], and also 0.93 with K6 in self-reported questionnaire [32]. Sakurai et al. reported the optimal cutoff point on K6 was 4/5 with sensitivity of 100% and specificity of 68.7% for screening mood and anxiety disorders in self-reported questionnaire [32]. This cutoff point of K6 score was comparable to Center for Epidemiologic Studies—Depression Scale (CES-D) with cutoff point of 15/16. Scored 16 and over in CES-D indicates clinical depression in Japan. CSLC included self-reported questionnaire with K6, therefore, this study employed cutoff point for 4 or 5 and over with K6 to assess non-specific psychological distress.

### Explanatory variables

The CSLC surveyed various characteristics of each individual and household. Therefore, we adjusted our empirical model by enriching certain sets of explanatory variables, such as sex, age, duration of care, and level of care needs (only for child) at the individual level and family structure (single parent, two parents, or three generation family), house ownership, and total household monthly expenditure at the household level. Note that we divided monthly household expenditure by the square root of the number of family members to standardize the effect of family size. We also used health-related conditions for both children and CGs as explanatory dummy variables as follows. The question for health care utilization was “Do you currently have regular outpatient visits to hospital, clinic, Japanese Traditional Massage, Acupuncture, Moxacautery, Judo-Orthopedics, or regular home visits by a physician?” Having regular visits to any kind of health care facility noted above was coded as ‘1’ for “yes” or otherwise ‘0’. The question for activity restriction was “Have you ever become bedridden, or experienced inability to conduct usual activity due to health problems, such as being absent from work or school or not being able to conduct housekeeping task, at least one day during last month?” Activity restriction was coded as ‘1’ for “yes” or otherwise ‘0’. Additionally, our model adjusted for other CG characteristics, such as educational achievement, occupational status, attending regular health check-ups, and having someone to consult with (hereafter referred to as ‘low social support’).

### Statistical Analysis

This study used K6 score as dependent variable with the cutoff point of 4/5. Scored 5 and over with K6 was coded as ‘1’ to express having psychological distress, or otherwise ‘0’. To examine the association between the psychological distress of the CG and the child, caregiver, and household characteristics, we performed Student’s t-test for continuous variables (age), the Fisher’s exact test for two variables (CG sex, having someone to consult with), and the χ^2^ test for other dichotomous variables (Child’s sex, disability level, duration of being in need of care, working status, hospital visits, activity restriction, family composition, having own house, and so on). Monthly household expenditure was used as a dichotomous variable, with a group encompassing households with expenditures in the lowest 25^th^ percentile and a second group encompassing those above the 25^th^ percentile. Furthermore, we conducted multivariable logistic regression with forced entry of the child, CG, and household variables after examining multicollinearity. We used STATA SE, version 13 (Stata Corp., College Station, TX, USA; 2013) for all analyses.

### Ethical Consideration

This study was approved by the official ethical review board of the University of Tsukuba (Document No.862, May/14/2014).

## Results

### Characteristics of child, parents, and household

Table 1 shows the child, parent (main CG), and household characteristics. The mean age of the children was 11.1±3.4 years old, and 64.9% of the children were boys. 11.3% of children were bedridden or stayed in a sitting position in their daily lives. A total of 73.6% of children had received more than five years of care, and the percentages of those children who required regular hospital visits or had activity restrictions were 51.2%, and 21.9%, respectively.

**Table 1:** Characteristics of children, their parent (main caregiver), and household.

Children (n = 549)	n (%)
Age (Mean, SD)	11.1, 3.4
(range)	(6–17)
Sex (Male)	356 (64.9)
Disability level	
1 Able to go out by oneself with supervision	233 (42.4)
2 Need assistance to go out	254 (46.3)
3 Able to maintain sitting position	23 (4.2)
4 Bedridden	39 (7.1)
Duration of being cared was more than 5 years	404 (73.6)
Visiting hospital regularly (yes)	281 (51.2)
Activity restriction due to own health condition (ie. school absence) in during last month (yes)	120 (21.9)
Parent (Main caregiver)	
Age(Mean, SD)	41.4, 5.6
(range)	(28–60)
Female	479 (87.3)
Graduation from junior high school or high school	257 (46.8)
Working status (yes)	267 (48.6)
Having a symptom in a few days	244(44.4)
Visiting hospital regularly (yes)	160 (29.1)
Visiting hospital due to mental health problem	17 (10.7)
Activity restriction due to own health condition (ie. job absence) in during last month (yes)	74 (13.5)
Total score of K6 (Median, IQR)	83 (15.1)
Scored 5 and over	4 (0–8)
Scored 13 and over	244 (44.4)
Not having someone to consult with	49 (8.9)
Not visited health checkups during last year	25 (4.6)
Smoking experience (Everyday, occasional, or previously)	254 (46.3)
Household	
Family composition:	
Single parent	39 (7.1)
Two parents	400 (72.9)
Three generation family	95 (17.3)
Other	15 (2.7)
House ownership	372 (67.8)
Monthly household expenditure‡ (median, IQR)	11.5 (9.0–15.0)
(range)	(1.6–173.5)

^‡^ ¥10,000 / the square root of person in household.

The mean age of the CGs was 41.4±5.6, and 87.3% of the CGs were mothers. Half of the CGs had graduated from junior or high school, and less than 50% of them were currently employed. The proportion of CGs who regularly visited hospitals was 29.1%; however, 10.7% of them were visiting hospitals due to mental health problems. Furthermore, 15.1% of the CGs had experienced activity restriction during the last month due to their own health problems. The total K6 score among the CGs indicated the prevalence of psychological distress; 44.4% of CGs had a K6 score of 5+, and 8.9% had a score of 13+, which indicates serious mental illness. Among the 549 included households, 7.1% were single-parent families, and most households contained two parents or three generations. The median monthly household expenditure was 115,000 yen, and it ranged from 16,000 to 1735,000 yen.

### Univariate analysis for CG psychological distress and related factors

Table 2 presents the univariate analysis of CG psychological distress and related factors. CG psychological distress was significantly correlated with child’s visiting hospital regularly (p = 0.007), mother as CG (p = 0.002), CG having symptom currently (p<0.001), CG’s visiting hospital regularly (p<0.001), CG having activity restriction (p<0.001), and low social support (p = 0.005).

**Table 2:** Association caregiver’s mental health and characteristics of children, caregiver, and households.

	K6 score < 5		K6 score > = 5		
	n	(%)	n	(%)	p
Child					
Age (n = 514) (mean, SD)	272	(11.3, 3.4)	244	(11.0, 3.3)	0.232
Sex (n = 516)					
Male	176	(52.2)	161	(47.8)	0.761
Female	96	(53.6)	83	(46.4)	
Disability level (n = 516)					
1: Able to go out with supervision	116	(53.7)	100	(46.3)	0.702
2–4: Need assistance to go out, Sitting position, or Bedridden	156	(52.0)	144	(48.0)	
Duration of being in need of care (n = 516)					
More than 1 year	72	(54.1)	61	(45.9)	0.703
More than 5 years	200	(52.2)	183	(47.8)	
Visiting hospital regularly (n = 494)					
No	135	(59.0)	94	(41.0)	**0.007**
Yes	124	(46.8)	141	(53.2)	
Activity restriction (n = 476)					
No	193	(53.6)	167	(46.4)	0.401
Yes	57	(49.1)	59	(50.9)	
Caregiver					
Age (n = 514) (mean, SD)	272	(41.2, 5.7)	244	(41.4, 5.5)	0.715
Sex (n = 516)					
Male	31	(75.6)	10	(24.4)	**0.002**
Female	241	(50.7)	234	(49.3)	
Education (n = 475)					
graduate from junior high, or high school	119	(49.2)	123	(50.8)	0.149
graduate from college, or others	130	(55.8)	103	(44.2)	
Working status (n = 514)					
No	139	(51.9)	129	(48.1)	0.753
Yes	131	(53.3)	115	(46.7)	
Having a symptom in a few days (n = 512)					
No	188	(66.9)	93	(33.1)	**<0.001**
Yes	81	(35.1)	150	(64.9)	
Visiting hospital regularly (n = 513)					
No	212	(58.7)	149	(41.3)	**<0.001**
Yes	575	(85.8)	95	(14.2)	
Activity restriction (n = 507)					
No	248	(58.1)	179	(41.9)	**<0.001**
Yes	21	(26.3)	59	(73.8)	
Having someone to consult with (n = 472)					
Yes	246	(54.9)	202	(45.1)	**0.005** ¶
No	2	(8.3)	22	(91.7)	
Visited health checkups during last year (n = 514)					
Yes	146	(54.3)	123	(45.7)	0.406
No	124	(50.6)	121	(49.4)	
Smoking experience (n = 515)					
Never	209	(52.1)	192	(47.9)	0.669
Every day, occasional, or previously	62	(54.4)	52	(45.6)	
Household					
Family composition (n = 516)					
Single parent, two parents	213	(51.2)	203	(48.8)	0.117
Three generation family	52	(60.5)	34	(39.5)	
Having own house (n = 516)					
Yes	192	(54.5)	160	(45.5)	0.222
No	80	(48.8)	84	(51.2)	
Monthly household expenditure ‡ (n = 516)					
Upper 25% tile and over	223	(51.0)	193	(49.0)	0.407
Lower 25% tile	49	(44.8)	51	(55.2)	

^‡^ ¥10,000 / the square root of numbers of person in household

χ^2 test

^¶^ Fisher exact test.

### Multivariable logistic regression for caregiver mental health

The results of the multivariable analysis are shown in Table 3. After adjusting for the child, CG, and household covariates, CG having a current symptom (OR, 95% CI: 3.26, 1.97–5.39), CG activity restriction (OR, 95% CI: 2.95, 1.38–6.32), low social support (OR, 95% CI: 9.31, 1.85–46.8), three generation family (OR, 95% CI: 0.49, 0.26–0.92), and being in the lower 25th percentile group for monthly household expenditure (OR, 95% CI: 1.92, 1.05–3.54) were significantly associated with CG psychological distress.

**Table 3:** Factors related to caregiver’s mental health (K6 ≧5) in multivariable logistic regression.

(n = 379)	OR	95%CI
Child		
Age	0.95	0.88–1.02
Sex (ref. male)	0.77	0.46–1.27
Disability level 2–4 (ref. level 1)	1.33	0.80–2.21
Longer cared duration (ref. <5 years)	1.20	0.69–2.09
Regular hospital visits (ref. no)	1.58	0.98–2.55
Had activity restriction (ref. no)	0.74	0.41–1.32
CG		
Sex (ref. male)	1.93	0.68–5.52
Working status (ref. no)	1.39	0.84–2.30
**Having current symptom (ref. no)**	**3.26**	**1.97–5.39**
Regular hospital visits (ref. no)	1.48	0.86–2.55
**Had activity restriction (ref. no)**	**2.95**	**1.38–6.32**
**Low social support (ref. yes)**	**9.31**	**1.85–46.83**
Had health checkups during last year. (ref. yes)	1.26	0.78–2.04
Never smoke (ref. having smoking experience)	1.06	0.57–1.99
Graduate from junior high school or high school (ref. junior college or above)	1.25	0.78–2.03
Household		
**Three generation family** (ref. one or two parents)	**0.49**	**0.26–0.92**
**Lowe 25%tile group of monthly household expenditure** ‡(ref. 75%tile of higher income group)	**1.92**	**1.05–3.54**

^‡^ ¥10,000 / the square root of numbers of person in household

Hosmer-Lemeshow gof: p = 0.374.

## Discussion

This study is the first to examine the types of factors that influence CG mental health using data obtained from a nationwide survey in Japan. We found that almost half of the CGs had psychological distress (K6 scored 5 and over) and that 8.9% of the CGs may have a serious mental illness (K6 score of 13 and above). Among the CGs, those who had any symptom currently, those who had experienced activity restriction, those who had lower social support, those who lived in single- or two-parent households, and those whose household expenditure belonged to the lowest 25th percentile were significantly more likely to have psychological distress.

The proportions of CGs with children with disabilities who had been suffering psychological distress and serious mental illness were higher than those for the overall Japanese population; in 2013, the Ministry of Health, Labor, and Welfare reported that 67.3% of community dwellers had a K6 score of 0 to 4, whereas only 2.6% of community dwellers had a K6 score of 15 or greater [33]. The mean and SD of the K6 scores of the CGs in the current study was 5.31±5.45. The mean K6 score of CGs of children with disabilities was higher than the mean score previously obtained for CGs of elderly persons, which Oshio reported to be 4.29±4.46 [34]. They used six-year panel data obtained from a nationwide population-based survey named “The Longitudinal Survey of Middle-Aged and Older Adults” and targeted CGs of 50–59 years of age, whose care recipients were most commonly their parents or parents-in-law. In another prior study [22], the mothers of children with pervasive developmental disabilities had a similar mean K6 score, 5.3±5.1, to that observed in the current study. Hence, much attention should be given to CGs with children with disabilities because they have a higher prevalence and severity of psychological distress than the overall population.

Currently having a symptom was another risk factor for psychological distress in CGs. Consistent with previous findings, severe somatic symptom was associated with increased psychological distress and health care utilization [35]. However, visiting a hospital was not significantly associated with psychological distress in the current study. Health care providers need to be aware of the possibility of CGs having psychological distress when they present with any subjective symptom, regardless of whether they visit a hospital.

Activity restriction was another risk factor for CG psychological distress. Of 52 CGs who had any type of activity restriction, half experienced 1–2 days of the restriction and nine (17.3%) experienced activity restrictions of over 7 days during the month prior to completing the survey. In this study, CGs were asked about their experiences with activity restrictions due to their own health problems. Physical health problems, mental health problems, or both could have caused the activity restriction. It is important to assess both the physical and mental health of CGs, and the effects of physical and mental health status on the social roles of CGs.

Being part of a three-generation family was a protective factor for CGs against the development of psychological distress. In previous studies, positive support from non-spousal family members also reduced the negative effects of the child’s disability on parental mental health [36], and informal support from a spouse, extended family members, or friends was associated with parental well-being [37]. Therefore, support from not only the spouse but also other family members is helpful for mothers who are taking care of a child with a disability. Ueda et al. [38] examined the mental health of Japanese CGs of children (over six years of age) with a disability. In her study, no support from the spouse or grandparents was a significant risk factor of poor mental health of the CG. Health care providers should be aware of the importance of social support from grandparents in addition to the spouse or partner and should ask CGs about the availability of social support from family members. In addition, the present study showed highest odds ratio (OR, 95%CI: 9.31, 1.85–46.8) for low social support (having no one to consult with). The lack of personal resource for CGs may have greatly affected their mental health. However, the current study did not examine what type of support, such as support from grandparents, friends, neighbors, or professionals, or what extent of support (frequency of access to the support) are the most protective against mental health problems in CGs. Further research should be performed to determine the most effective support in both qualitative and quantitative aspects.

Lower monthly expenditure, which was a surrogate variable for lower income, was significantly correlated with psychological distress in the current study. Because children with disabilities usually utilize higher health care services more frequently than other children [39], they tend to have much higher health care expenditures and out-of-pocket expenditures [40]. Furthermore, family members may have to decrease their working hours or stop working to care for the child, especially a child with a more medically complex condition [41]. Thus, lower income families with disabled children may have greater financial burdens leading to parental psychological distress. However, another possible relationship between low income and parental psychological distress should be considered because this study was cross-sectional. Socioeconomic disadvantage may be a consequence of raising a child with a disability, or the cause of the disability. Spencer and Strazdins [42] performed a cohort study and found that socioeconomic disadvantage preceded the onset of chronic disabling conditions in children. Families with lower income tend to experience psychosocial and physical stressors, such as family turmoil, violence, and house problems [43]. Therefore, the CGs of lower income families in the present study may have experienced psychological distress before the onset of the child’s disability, or the onset of the child’s disability may have exacerbated the CG’s psychological distress that resulted from caring for the child. Future longitudinal studies should examine the changes in psychological distress among CGs overtime, and health professionals should pay attention to the support CGs receive to decrease their psychological distress in regards to supporting the child’s health development.

### Limitations and Future Directions

The current study has several limitations. First, the information regarding children’s characteristics was limited. This study focused on children with disabilities in regards to severity of care needs; however, the questionnaire did not include the diagnoses of diseases or disabilities, the severity of behavioral problems, or the child’s communication abilities. The children may have had cerebral palsy, congenital heart disease, autism, or a learning disability. Furthermore, we did not obtain information on medical care at home, such as medications, tubal feeding, and suctioning. Future studies should collect a more detailed medical history in regards to the underlying diseases and medical care required at home. Second, except for regular hospital visits, this study was unable to examine health care service use, such as home-visit nursing care, physician home-visit, rehabilitation service at home or at a hospital, and acute hospitalizations or emergency department visits. Home health services may have protective effects on CG mental health by maintaining the child’s health regularly, and emergent health care use may indicate the level of difficulty of taking care of the child. Finally, the study sample size was relatively small, though the current study only targeted children who had mild to severe restrictions in activities of daily living and the data were obtained from a national representative survey. It is necessary to evaluate the actual conditions of children with disabilities and families. The National Survey of Children With Special Health Care Needs was a nationwide survey conducted in the US that examined medical care use and unmet medical care needs [41]. The current study used LSLC, which aimed to examine the living conditions of Japanese families; however, it did not focus on children with disabilities and their families. Although the survey on persons with physical disabilities conducted by the Ministry of Health, Labor, and Welfare [1] targeted children and adults with disabilities, it did not collect detailed information on medical care use or CG characteristics. A nationwide survey of children with disabilities and CGs is urgently needed to support the health of both disabled children and their families in Japan. The Carers Act was issued in 2014 by the United Kingdom and clearly states that local authority has the duty of assessing the carer’s needs for supporting adults and children with disabilities [44]. In the United States, the American Academy of Pediatrics launched the Task Force on the Family and proposed several recommendations to assist families in functioning well and meeting the children’s needs [45]. Family support is vital for promoting the child health care system and protecting the health and rights of CGs. Further attention should be paid toward the effects of health problems of CGs on the health of their disabled child and to the rights of CGs to live healthy and participate in society.

## Conclusion

Half of parents taking care of children with disabilities experience psychological distress in Japan. This study encourages health care providers to pay more attention to the mental health of CGs, especially for CGs with younger children, having health problems of their own, activity restrictions, or low social support and for CGs of lower income families. Further research should examine more detailed information regarding the disease and disability of disabled children, their medical service use, and the quality and quantity of the CGs’ social support to improve the method of providing supporting service for both children with disabilities and their families.

## References

[1] The Ministory of Health Labour and Welfare. Survey on persons with physical disability, 2011. 2013 [cited 2015 March 31]. Available from: http://www.mhlw.go.jp/toukei/list/dl/seikatsu_chousa_b_h23.pdf.

[2] Statistics Bureau, Ministry of Internal Affairs and Communications Japan. Final report of the 2010 Population Census 2014 [cited 2015 24/06]. Available from: http://www.stat.go.jp/english/data/kokusei/2010/final_en/final_en.htm.

[3] The Ministry of Education Science Sports and Culture. The surevey for the children in regular class who have possibilities of developmental disability and need special education 2012 [cited 2015 march 30]. Available from: http://www.mext.go.jp/a_menu/shotou/tokubetu/material/__icsFiles/afieldfile/2012/12/10/1328729_01.pdf.

[4] Horiuchi T, Ohno T, Itani Y, Kabe K, Nakamura T, Nakamura H. Studies on the State of Care for High Risk Neonates and Neonatal Mortality in our country (year 2000). The Journal of the Japan Pediatric Society. 2006;106(4):603–13.

[5] Uetani Y. Nationwide survey for development at age of six among children born in 2005 with extremely low birth weight 2012 [cited 2015 05/10]. Available from: https://mhlw-grants.niph.go.jp/niph/search/NIDD00.do?resrchNum=201219006B.

[6] Ministory of Health Labour and Welfare. Vital Statistics 2014 2014 [cited 2015 05/10]. Available from: http://www.e-stat.go.jp/SG1/estat/List.do?lid=000001137964.

[7] Okamoto R. Association with increase of children with intellectual disability and maternal age Journal of Health and Welfare Statistics. 2014;12:1–7.

[8] Tsuchiya KJ, Matsumoto K, Miyachi T, Tsujii M, Nakamura K, Takagai S, et al Paternal age at birth and high-functioning autistic-spectrum disorder in offspring. The British journal of psychiatry: the journal of mental science. 2008;193(4):316–21. Epub 2008/10/02. 10.1192/bjp.bp.107.045120 . 18827294

[9] Raina P, O'Donnell M, Schwellnus H, Rosenbaum P, King G, Brehaut J, et al Caregiving process and caregiver burden: conceptual models to guide research and practice. BMC pediatrics. 2004;4:1 Epub 2004/01/16. 10.1186/1471-2431-4-1 ; PubMed Central PMCID: PMCPmc331415. 14723791 PMC331415

[10] Raina P, O'Donnell M, Rosenbaum P, Brehaut J, Walter SD, Russell D, et al The health and well-being of caregivers of children with cerebral palsy. Pediatrics. 2005;115(6):e626–36. Epub 2005/06/03. 10.1542/peds.2004-1689 . 15930188

[11] Parkes J, Caravale B, Marcelli M, Franco F, Colver A. Parenting stress and children with cerebral palsy: a European cross-sectional survey. Developmental medicine and child neurology. 2011;53(9):815–21. Epub 2011/06/29. 10.1111/j.1469-8749.2011.04014.x . 21707599

[12] Estes A, Olson E, Sullivan K, Greenson J, Winter J, Dawson G, et al Parenting-related stress and psychological distress in mothers of toddlers with autism spectrum disorders. Brain & development. 2013;35(2):133–8. Epub 2012/11/14. 10.1016/j.braindev.2012.10.004 ; PubMed Central PMCID: PMCPmc3552060. 23146332 PMC3552060

[13] Montes G, Halterman JS. Association of childhood autism spectrum disorders and loss of family income. Pediatrics. 2008;121(4):e821–6. Epub 2008/04/03. 10.1542/peds.2007-1594 . 18381511

[14] Lindley LC, Mark BA. Children with special health care needs: Impact of health care expenditures on family financial burden. Journal of child and family studies. 2010;19(1):79–89. 10.1007/s10826-009-9286-6 ; PubMed Central PMCID: PMCPmc2872488. 20495615 PMC2872488

[15] Parish SL, Rose RA, Dababnah S, Yoo J, Cassiman SA. State-level income inequality and family burden of U.S. families raising children with special health care needs. Social science & medicine (1982). 2012;74(3):399–407. Epub 2011/12/24. 10.1016/j.socscimed.2011.10.035 . 22192773

[16] Montes G, Halterman JS. Child care problems and employment among families with preschool-aged children with autism in the United States. Pediatrics. 2008;122(1):e202–8. Epub 2008/07/04. 10.1542/peds.2007-3037 . 18595965

[17] Nes RB, Hauge LJ, Kornstad T, Kristensen P, Landolt MA, Eskedal LT, et al The Impact of Child Behaviour Problems on Maternal Employment: A Longitudinal Cohort Study. Journal of family and economic issues. 2014;35:351–61. Epub 2014/08/29. 10.1007/s10834-013-9378-8 ; PubMed Central PMCID: PMCPmc4141146. 25165417 PMC4141146

[18] The Ministry of Health Labour and Welfare. Comprehensive Survey of Living Conditions, 2010 2011 [cited 2015 March 30]. Available from: http://www.mhlw.go.jp/toukei/saikin/hw/k-tyosa/k-tyosa10/dl/gaikyou.pdf.

[19] Statistics Bureau MoIAaC,. 2011 Survey on Time Use and Leisure Activities (Table 14) 2012 [cited 2015 05/10]. Available from: http://www.stat.go.jp/data/shakai/2011/gaiyou.htm.

[20] Ferro MA, Speechley KN. Depressive symptoms among mothers of children with epilepsy: a review of prevalence, associated factors, and impact on children. Epilepsia. 2009;50(11):2344–54. Epub 2009/08/22. 10.1111/j.1528-1167.2009.02276.x . 19694788

[21] Singer GH. Meta-analysis of comparative studies of depression in mothers of children with and without developmental disabilities. American journal of mental retardation: AJMR. 2006;111(3):155–69. Epub 2006/04/07. 10.1352/0895-8017(2006)111[155:mocsod]2.0.co;2 . 16597183

[22] Yamada A, Suzuki M, Kato M, Suzuki M, Tanaka S, Shindo T, et al Emotional distress and its correlates among parents of children with pervasive developmental disorders. Psychiatry and clinical neurosciences. 2007;61(6):651–7. Epub 2007/12/18. 10.1111/j.1440-1819.2007.01736.x . 18081627

[23] Goodman SH, Rouse MH, Connell AM, Broth MR, Hall CM, Heyward D. Maternal depression and child psychopathology: a meta-analytic review. Clinical child and family psychology review. 2011;14(1):1–27. Epub 2010/11/06. 10.1007/s10567-010-0080-1 . 21052833

[24] Lovejoy MC, Graczyk PA, O'Hare E, Neuman G. Maternal depression and parenting behavior: a meta-analytic review. Clinical psychology review. 2000;20(5):561–92. Epub 2000/06/22. . 10860167 10.1016/s0272-7358(98)00100-7

[25] Phelan KJ, Morrongiello BA, Khoury JC, Xu Y, Liddy S, Lanphear B. Maternal supervision of children during their first 3 years of life: the influence of maternal depression and child gender. Journal of pediatric psychology. 2014;39(3):349–57. Epub 2013/12/21. 10.1093/jpepsy/jst090 ; PubMed Central PMCID: PMCPmc3959264. 24357732 PMC3959264

[26] Yamaguchi S, Takataya K, Ogiwara T. Study of Mental Health and its Related Factors including Burden on Caregivers of Children/Persons with Severe Motor and Intellectual Disabilities. Yamanashi Nursing Journal. 2005;4(1):41–7.

[27] Yatsugi S, Suzukamo Y, Izumi S. [Productive social activities in mothers of intellectually disabled children moderate the relationship between caregiver burden and self-rated health]. [Nihon koshu eisei zasshi] Japanese journal of public health. 2013;60(7):387–95. Epub 2013/10/11. . 24107302

[28] Toki M, Washio M, Furukawa A, Narita H, Yokogushi K, Ishiai S. Investigating Parental Caregiver Burden for Children with Disabilities using a Japanese version of the Zarit Caregiver Burden Interview (J-ZBI). Jpn J Rehabil Med. 2010;47:396–404.

[29] Kessler RC, Green JG, Gruber MJ, Sampson NA, Bromet E, Cuitan M, et al Screening for serious mental illness in the general population with the K6 screening scale: results from the WHO World Mental Health (WMH) survey initiative. International journal of methods in psychiatric research. 2010;19 Suppl 1:4–22. Epub 2010/06/08. 10.1002/mpr.310 ; PubMed Central PMCID: PMCPmc3659799. 20527002 PMC3659799

[30] Kessler RC, Andrews G, Colpe LJ, Hiripi E, Mroczek DK, Normand SL, et al Short screening scales to monitor population prevalences and trends in non-specific psychological distress. Psychological medicine. 2002;32(6):959–76. Epub 2002/09/07. . 12214795 10.1017/s0033291702006074

[31] Furukawa TA, Kawakami N, Saitoh M, Ono Y, Nakane Y, Nakamura Y, et al The performance of the Japanese version of the K6 and K10 in the World Mental Health Survey Japan. International journal of methods in psychiatric research. 2008;17(3):152–8. Epub 2008/09/04. 10.1002/mpr.257 . 18763695 PMC6878390

[32] Sakurai K, Nishi A, Kondo K, Yanagida K, Kawakami N. Screening performance of K6/K10 and other screening instruments for mood and anxiety disorders in Japan. Psychiatry and clinical neurosciences. 2011;65(5):434–41. Epub 2011/08/20. 10.1111/j.1440-1819.2011.02236.x . 21851452

[33] The Ministry of Health Labour and Welfare. Comprehensive Survey of Living Conditions, 2013 2013 [cited 2015 05/24]. Available from: http://www.mhlw.go.jp/toukei/saikin/hw/k-tyosa/k-tyosa13/index.html.

[34] Oshio T. The association between involvement in family caregiving and mental health among middle-aged adults in Japan. Social science & medicine (1982). 2014;115:121–9. Epub 2014/06/24. 10.1016/j.socscimed.2014.06.016 . 24955876

[35] Lee S, Creed FH, Ma YL, Leung CM. Somatic symptom burden and health anxiety in the population and their correlates. Journal of psychosomatic research. 2015;78(1):71–6. Epub 2014/12/04. 10.1016/j.jpsychores.2014.11.012 . 25466323

[36] Ha JH, Greenberg JS, Seltzer MM. Parenting a Child with a Disability: The Role of Social Support for African American Parents. Families in society: the journal of contemporary human services. 2011;92(4):405–11. Epub 2011/01/01. 10.1606/1044-3894.4150 ; PubMed Central PMCID: PMCPmc3364020. 22661878 PMC3364020

[37] White N, Hastings RP. Social and Professional Support for Parents of Adolescents with Severe Intellectual Disabilities. Journal of Applied Research in Intellectual Disabilities. 2004;17(3):181–90. 10.1111/j.1468-3148.2004.00197.x

[38] Ueda K, Mbumba FN, Mori R, Nakamura Y, Kitajima H, Okamoto N. Mental Health of Families of Pediatric Outpatients as Reflected in the 28-item General Health Questionnaire Japanese Version (GHQ28). The Journal of the Japan Pediatric Society. 2010;114(9):1419–26.

[39] Newacheck PW, Strickland B, Shonkoff JP, Perrin JM, McPherson M, McManus M, et al An epidemiologic profile of children with special health care needs. Pediatrics. 1998;102(1 Pt 1):117–23. Epub 1998/07/04. . 9651423 10.1542/peds.102.1.117

[40] Newacheck PW, Inkelas M, Kim SE. Health services use and health care expenditures for children with disabilities. Pediatrics. 2004;114(1):79–85. Epub 2004/07/03. . 15231911 10.1542/peds.114.1.79

[41] Kuo DZ, Cohen E, Agrawal R, Berry JG, Casey PH. A national profile of caregiver challenges among more medically complex children with special health care needs. Archives of pediatrics & adolescent medicine. 2011;165(11):1020–6. Epub 2011/11/09. 10.1001/archpediatrics.2011.172 ; PubMed Central PMCID: PMCPmc3923457. 22065182 PMC3923457

[42] Spencer N, Strazdins L. Socioeconomic disadvantage and onset of childhood chronic disabling conditions: a cohort study. Archives of disease in childhood. 2015;100(4):317–22. Epub 2014/09/23. 10.1136/archdischild-2013-305634 . 25239950

[43] Evans GW, English K. The environment of poverty: multiple stressor exposure, psychophysiological stress, and socioemotional adjustment. Child development. 2002;73(4):1238–48. Epub 2002/07/31. . 12146745 10.1111/1467-8624.00469

[44] The Stationery Office. Care Act 2014 2014 [cited 2015 05/19]. Available from: http://www.legislation.gov.uk/ukpga/2014/23/pdfs/ukpga_20140023_en.pdf.

[45] Schor EL. Family pediatrics: report of the Task Force on the Family. Pediatrics. 2003;111(6 Pt 2):1541–71. Epub 2003/06/05. . 12777595

